# Supplementary material for: Global and local ancestry modulate APOE association with Alzheimer’s neuropathology and cognitive outcomes in an admixed sample
Source: Mol Psychiatry. 2022 Sep 7;27(11):4800–8. doi: 10.1038/s41380-022-01729-x (PMC9734036; doi:10.1038/s41380-022-01729-x)
Supplement: Supplementary file 1 — Supplementary Material [file 41380_2022_1729_MOESM1_ESM.docx]

**Supplementary Information**

**Global and local ancestry modulate *APOE* association with Alzheimer's neuropathology and cognitive outcomes in an admixed sample**

**Supplementary methods**

**DNA extraction**

DNA samples were extracted from post-mortem cardiac blood samples using Gentra Autopure LS automated protocol, followed by measurement of DNA quality and concentration spectrophotometer, as previously described (1). If DNA parameters have not reached minimum standards for genetic analyses, DNA was isolated and purified from frozen cerebellum using manual standard salt and EDTA cell lysis followed by phenol-chloroform isoamyl alcohol extraction yielding adequate samples for subsequent analyses.

**Global ancestry inference**

BAS samples used in this study were subjected to either one or both of the following genotyping methods. A total of 724 individuals were genotyped using Illumina Human OmniExpress 700k microarray, of which 309 were included in this study. The remaining 91 individuals were genotyped using the Illumina BeadXpress platform, using a custom panel containing ancestry informative markers (AIMs) used to calculate global ancestry (2). To maximize the number of samples and avoid the imputation of the non-overlapping variants, global ancestry inference was performed with genotypes of the overlapping 47 SNVs. The global ancestry correlation of this subset of markers and the full panel was 0.94 after verification using 246 samples that were genotyped using both the microarray and the custom BeadXpress panel.

We inferred sample structure with Structure 2.3.4 (3). The run was performed assuming K=3 populations, based on the main parental population groups that originated modern Brazilians (4); additional run parameters included a burn-in procedure of 100,000 steps, followed by 100,000 Markov Chain Monte Carlo iterations, using the admixture model with allele frequency correlated among populations. To assist ancestry inference, we included genotypes of individuals from reference populations obtained from the HapMap Project (Central Europeans from Utah, n=57; Yoruba in Ibadan, Nigeria, n=62) and Kosoy et al. (2009) (2); 188 European descent from Utah and New York, 98 West African individuals from Nigeria and Niger-Congo region and 105 Native American descent individuals from Guatemala, Peru, and Mexico).

***APOE* genotyping, imputation, and local ancestry inference**

*APOE* common alleles are composed of a combination of genotypes in variants rs7412 and rs429358, the latter of which is absent from the microarray. Imputation was performed using a window from the genotyping array dataset composed of 724 individuals (including the 309 used in this study) to determine the rs429358 genotype. The window included 2.216 SNPs in a ~10Mb (11.323.329 bp) region from 40.404.633 bp to 51.727.962 bp from chromosome 19, which consists of the rs7412 (19:45412079) but not the rs429358 (19:45411941) in GRCh37 assembly. This region has been selected to focus on ~±5 Mb around the rs7412 interest.

We imputed the target dataset with a reference panel that merged the public reference panel data from 1000 Genomes Phase 3 Project (1KGP) and 270 individuals from EPIGEN-Brazil (90 of each cohort) genotyped for 4.3 million markers, the EPIGEN-5M+1KGP imputation panel, fully described in Magalhães et al. (2018) (5) and considered only SNPs imputed with an info score quality metric > 0.8. All imputation tasks detailed below were performed using the master script described elsewhere (5). Pre-phasing between the target and reference panels has been done using SHAPEIT2 (6) to check the consistency of the marker's strand of target and reference panels with the human genome reference sequence and PLINK software (7) to flip the strands in case of inconsistencies. Then, haplotype phase inference of the target dataset was made using the 1KGP haplotypes. Finally, imputation has been done using the EPIGEN-5M+1KGP imputation panel and IMPUTE2 v.2.3.2 software (8) on chromosome chunks of 7 Mb, with effective size parameter (Ne), set to 20,000 and the IMPUTE2 info score as a metric of imputation quality. QCTOOL (Available at: https://www.well.ox.ac.uk/~gav/qctool/) has been used for data quality control, filtering, and conversions.

Imputation performance was verified with direct *APOE* genotyping obtained using allele-specific amplification and real-time PCR, as described previously (9), which was conducted orthogonally in BAS samples, including most that were genotyped using microarray and custom panel. For a total of 523 cases that overlapped *APOE* genotyping methods, we obtained a 93.3% accuracy. We chose the result from direct genotyping in all samples available. Among the 309 individuals with genotyped array included in this study, a total of 28 cases were APOE genotyped using imputation of rs429358.

For local ancestry inference (LAI), we selected Africans, Europeans, and Native Americans from Public datasets (1000Genomes Project (10) and Human Genome Diversity Project [HGDP] (11) WGS). Specifically, from 1000 Genomes, we selected YRI and LWK as African references, CEU and IBS as European. For the Native American ancestry, we selected individuals with a high proportion of Native American Ancestry from PEL and MXL and Native Americans from HGDP, in addition to other Native American samples recently published (12).

We selected the genomic region corresponding to the *APOE* gene (GRCh37, chromosome 19 from 40416921 to 51727962, ~11Mb), including 2,234 SNPs in our dataset. We merged with the public datasets using PLINK, generating a dataset with 1349 individuals. We phased the merged data using SHAPEIT2(6). We applied RFMix (13) using two Expectation-Maximization iterations with a minimum node size of 5. Only inferred local ancestries per haplotype with 80% or above posterior probabilities were included.

**Neuropathological assessment**

The BAS-USP follows standardized protocols. Brain tissue is obtained within 24 hours of death (14, 15). One hemisphere is fixed in buffered paraformaldehyde. After fixation, samples from the following selected areas are embedded in paraffin: middle frontal gyrus, middle, and superior temporal gyri, angular gyrus, superior frontal, and anterior cingulate gyrus, visual cortex, hippocampal formation at the level of the lateral geniculate body, amygdala, basal ganglia at the level of the anterior commissure, thalamus, midbrain, pons, medulla oblongata, and cerebellum. Immunohistochemistry of selected sections is performed using antibodies against beta-amyloid (4G8), phosphorylated tau (CP-13), α-synuclein (MDV), and TDP-43 (Proteintech) (15). The pathologists (LTG and RDR) conduct analysis blinded to demographics and clinical outcomes. Both hemispheres undergo gross assessment. Microvascular changes were analyzed semi-quantitatively using hematoxylin and eosin staining in all sampled areas. Small-vessel disease is diagnosed when there is widespread (more than 50%) and at least moderate small-vessel disease in white matter in at least three cortical regions out of those examined (superior frontal gyrus, medium frontal gyrus, inferior and medium temporal gyri, anterior cingulate gyrus, angular gyrus), and includes small-vessel arteriosclerosis/atherosclerosis, arteriolosclerosis, and lipohyalinosis (15). Cases are classified as positive for cerebral amyloid angiopathy (CAA) when CAA is presently diffusely in the parenchyma of at least three different cortical areas (16) Inclusion in the group "infarct" requires one large chronic infarct (>1 cm) or three lacunae in strategic areas - thalamus, frontocingular cortex, basal forebrain and caudate, medial temporal area, and angular gyrus (16, 17). Cases meeting criteria for small vessel disease or infarcts were excluded from the analysis. AD-type neuropathology is evaluated using the Consortium to Establish a Registry for AD (CERAD) criteria for neuritic plaque burden (18)), and the Braak and Braak staging for neurofibrillary tangle pathology (19)

Lewy-type pathology is assessed using the Braak staging for Parkinson's disease (20), and groups were divided as Lewy body disease present (Braak≥0) or absent. Cases with amygdala-predominant pathology are noted (21). Cases with any level of Lewy-body pathology were excluded from the analysis.TDP-43 pathology is assessed in amygdala and hippocampus (22). If positive, the workup is extended to several neocortical areas. Cases with any level of TDP-43 proteinopathy were excluded from the analysis.

**References**

1. Schlesinger D, Grinberg LT, Alba JG, Naslavsky MS, Licinio L, Farfel JM, et al. African ancestry protects against Alzheimer's disease-related neuropathology. Mol Psychiatry. 2013;18(1):79-85.

2. Kosoy R, Nassir R, Tian C, White PA, Butler LM, Silva G, et al. Ancestry informative marker sets for determining continental origin and admixture proportions in common populations in America. Hum Mutat. 2009;30(1):69-78.

3. Falush D, Stephens M, Pritchard JK. Inference of population structure using multilocus genotype data: dominant markers and null alleles. Mol Ecol Notes. 2007;7(4):574-8.

4. Salzano FM, Sans M. Interethnic admixture and the evolution of Latin American populations. Genet Mol Biol. 2014;37(1 Suppl):151-70.

5. Magalhaes WCS, Araujo NM, Leal TP, Araujo GS, Viriato PJS, Kehdy FS, et al. EPIGEN-Brazil Initiative resources: a Latin American imputation panel and the Scientific Workflow. Genome Res. 2018;28(7):1090-5.

6. Delaneau O, Zagury JF, Marchini J. Improved whole-chromosome phasing for disease and population genetic studies. Nat Methods. 2013;10(1):5-6.

7. Purcell S, Neale B, Todd-Brown K, Thomas L, Ferreira MA, Bender D, et al. PLINK: a tool set for whole-genome association and population-based linkage analyses. Am J Hum Genet. 2007;81(3):559-75.

8. Howie BN, Donnelly P, Marchini J. A flexible and accurate genotype imputation method for the next generation of genome-wide association studies. PLoS Genet. 2009;5(6):e1000529.

9. Calero O, Hortiguela R, Bullido MJ, Calero M. Apolipoprotein E genotyping method by real time PCR, a fast and cost-effective alternative to the TaqMan and FRET assays. J Neurosci Methods. 2009;183(2):238-40.

10. Genomes Project C, Auton A, Brooks LD, Durbin RM, Garrison EP, Kang HM, et al. A global reference for human genetic variation. Nature. 2015;526(7571):68-74.

11. Bergstrom A, McCarthy SA, Hui R, Almarri MA, Ayub Q, Danecek P, et al. Insights into human genetic variation and population history from 929 diverse genomes. Science. 2020;367(6484).

12. Scliar MO, Sant'Anna HP, Santolalla ML, Leal TP, Araujo NM, Alvim I, et al. Admixture/fine-mapping in Brazilians reveals a West African associated potential regulatory variant (rs114066381) with a strong female-specific effect on body mass and fat mass indexes. Int J Obes (Lond). 2021;45(5):1017-29.

13. Maples BK, Gravel S, Kenny EE, Bustamante CD. RFMix: a discriminative modeling approach for rapid and robust local-ancestry inference. Am J Hum Genet. 2013;93(2):278-88.

14. Grinberg LT, Ferretti RE, Farfel JM, Leite R, Pasqualucci CA, Rosemberg S, et al. Brain bank of the Brazilian aging brain study group - a milestone reached and more than 1,600 collected brains. Cell Tissue Bank. 2007;8(2):151-62.

15. Suemoto CK, Ferretti-Rebustini RE, Rodriguez RD, Leite RE, Soterio L, Brucki SM, et al. Neuropathological diagnoses and clinical correlates in older adults in Brazil: A cross-sectional study. PLoS Med. 2017;14(3):e1002267.

16. Grinberg LT, Thal DR. Vascular pathology in the aged human brain. Acta Neuropathol. 2010;119(3):277-90.

17. Jellinger KA, Attems J. Prevalence and impact of cerebrovascular pathology in Alzheimer's disease and parkinsonism. Acta Neurol Scand. 2006;114(1):38-46.

18. Mirra SS, Heyman A, McKeel D, Sumi SM, Crain BJ, Brownlee LM, et al. The Consortium to Establish a Registry for Alzheimer's Disease (CERAD). Part II. Standardization of the neuropathologic assessment of Alzheimer's disease. Neurology. 1991;41:479-86.

19. Braak H, Braak E. Neuropathological stageing of Alzheimer-related changes. Acta Neuropathol. 1991;82(4):239-59.

20. Braak H, Del Tredici K, Rub U, de Vos RA, Jansen Steur EN, Braak E. Staging of brain pathology related to sporadic Parkinson's disease. Neurobiol Aging. 2003;24(2):197-211.

21. Attems J, Toledo JB, Walker L, Gelpi E, Gentleman S, Halliday G, et al. Neuropathological consensus criteria for the evaluation of Lewy pathology in post-mortem brains: a multi-centre study. Acta Neuropathol. 2021;141(2):159-72.

22. Nelson PT, Dickson DW, Trojanowski JQ, Jack CR, Boyle PA, Arfanakis K, et al. Limbic-predominant age-related TDP-43 encephalopathy (LATE): consensus working group report. Brain. 2019;142(6):1503-27.

**Supplementary Figures**

**
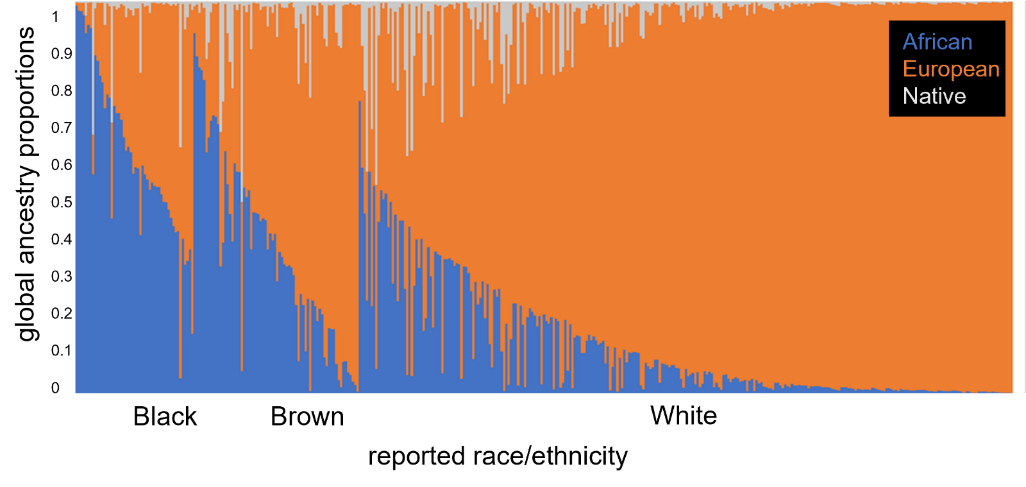
**

**Supplementary Figure 1.** Distribution of global ancestry proportions vs. reported race/ethnicity in the study population (N=397, three individuals of Asian reported race/ethnicity not shown in graph).

**
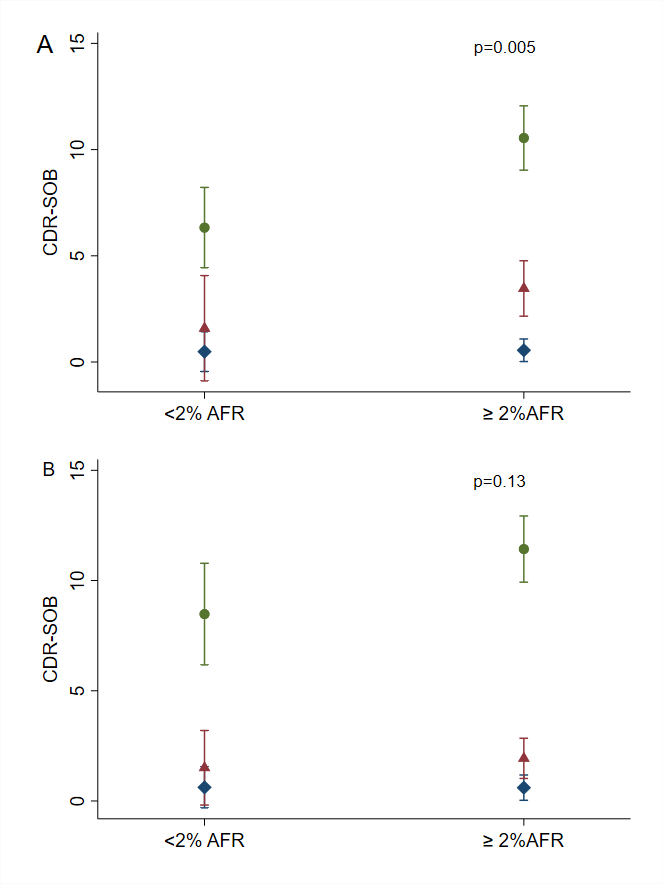
**

**Supplementary Figure 2**. Association between African ancestry and Clinical Dementia Rating Sum of Boxes (CDR-SOB) considering an interaction term between African ancestry and neuritic plaques evaluated by the CERAD score (**A**), or neurofibrillary tangle burden evaluated by the Braak & Braak stage (**B**). (**A**) neuritic plaque burden in all individuals [Blue diamond: None or sparse (n=303); Red triangle: Moderate (n=49); Green circle: Frequent (n=48)]; (**B**) neurofibrillary tangle burden in all individuals [Blue diamond: 0-II (n=263); Red triangle: III-IV (n=99); Green circle: V-VI (n=38)]. P-values for the interaction terms included in linear regression models adjusted for age, sex, education, and *APOE4* status (A and B). Neuritic plaques were evaluated by the Consortium to Establish a Registry for Alzheimer's disease (CERAD) score, and neurofibrillary tangles were evaluated by the Braak staining system.

**
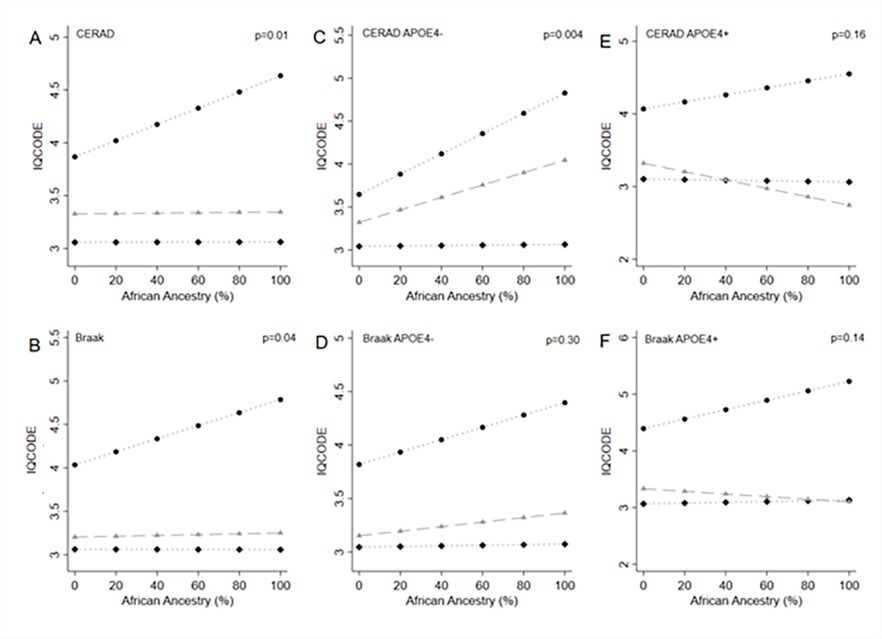
**

**Supplementary Figure 3.** Association between African ancestry and Informant Questionnaire on Cognitive Decline in the Elderly (IQCODE) considering an interaction term between African ancestry and neuritic plaques evaluated by the CERAD score (**A, C, and E**), or neurofibrillary tangle burden evaluated by the Braak & Braak stage (**B, D, and F**). (**A**) neuritic plaque burden in all individuals [Black diamond: None or sparse (n=303); Grey triangle: Moderate (n=49); Black circle: Frequent (n=48)]; (**B**) neurofibrillary tangle burden in all individuals [Black diamond: 0-II (n=263); Grey triangle: III-IV (n=99); Black circle: V-VI (n=38)]; (**C**) neuritic plaques in *APOE*4- individuals [Black diamond: None or sparse plaques (n=239); Grey triangle: Moderate plaques (n=31); Black circle: Frequent plaques (n=22)]; (**D**) neurofibrillary tangles in *APOE*4- individuals [Black diamond: Braak 0-II (n=206); Grey triangle: Braak III-IV (n=64); Black circle: Braak V-VI (n=22)]; (**E**) neuritic plaques in *APOE*4+ individuals [Black diamond: None or sparse plaques (n=64); Grey triangle: Moderate plaques (n=18); Black circle: Frequent plaques (n=26); and (**F**) neurofibrillary tangles in *APOE*4+ individuals [Black diamond: Braak 0-II (n=57); Grey triangle: Braak III-IV (n=35); Black circle: Braak V-VI (n=16)]. P-values for the interaction terms included in linear regression models adjusted for age, sex, education, and *APOE4* status (A and B). Neuritic plaques were evaluated by the Consortium to Establish a Registry for Alzheimer's disease (CERAD) score, and neurofibrillary tangles were evaluated by the Braak staining system.

**Supplementary Tables**

**Supplementary Table 1. Characteristics of individuals that were reported as White according to African ancestry (n=277)**

|  | < 2%AFR  n=97 | ≥ 2%AFR  n=180 | p |
| --- | --- | --- | --- |
| Age (years), mean (SD)* | 74.0 (10.9) | 72.5 (12.8) | 0.35 |
| Female, %^†^ | 46.4 | 49.4 | 0.63 |
| Education (years), mean (SD)* | 6.0 (4.0) | 4.4 (3.8) | 0.001 |
| CDR ≥ 0.5, %^†^ | 18.6 | 12.2 | 0.15 |
| CDR sum of boxes, mean (SD)* | 1.7 (4.6) | 1.6 (4.7) | 0.83 |
| At least one APOE ε4, %^†^ | 24.7 | 23.3 | 0.79 |
| Braak & Braak score, %^†^ |  |  | 0.40 |
| *0-II* | 67.0 | 63.3 |  |
| *III-IV* | 21.7 | 28.3 |  |
| *V-VI* | 11.3 | 8.4 |  |
| CERAD score, %^†^ |  |  | 0.09 |
| *None or Sparse* | 71.1 | 74.4 |  |
| *Moderate* | 10.3 | 15.6 |  |
| *Frequent* | 18.6 | 10.0 |  |

AFR: global African ancestry; SD: standard deviation; CERAD: Consortium to Establish a Registry for Alzheimer's disease

*unpaired t-test; ^‡^Fisher exact test; ^†^chi-square test

**Supplementary Table 2**. Distribution of ancestry proportions across reported race/ethnicity groups

|  |  | **Ancestry proportions (average ± stdev [min-max])** | | |
| --- | --- | --- | --- | --- |
| **Reported race/ethnicity** | **N*** | **EUR** | **AFR** | **NAT** |
| **Black** | 50 | 0.36 ± 0.2 [0.01-0.83] | 0.61 ± 0.22 [0.05-0.99] | 0.04 ± 0.09 [0.01-0.38] |
| **Brown** | 70 | 0.59 ± 0.24 [0.07-0.99] | 0.36 ± 0.25 [0.01-0.92] | 0.06 ± 0.09 [0.01-0.51] |
| **White** | 277 | 0.86 ± 0.17 [0.25-1] | 0.11 ± 0.14 [0.01-0.75] | 0.04 ± 0.08 [0.01-0.47] |
| **All** | 397 | 0.75 ± 0.26 [0.01-1] | 0.22 ± 0.25 [0.01-0.99] | 0.05 ± 0.09 [0.01-0.51] |

*Three samples reported as Asians were excluded from the ancestry vs. reported race/ethnicity distribution description

**Supplementary Table 3. Association between dichotomous African ancestry* and AD neuropathological burden (n=400)**

|  | Crude |  | Model 1 |  | Model 2 |  |
| --- | --- | --- | --- | --- | --- | --- |
|  | **OR (95% CI)** | **p** | **OR (95% CI)** | **p** | **OR (95% CI)** | **p** |
| BB stage | 1.13 (0.75; 1.69) | 0.56 | 1.36 (0.89; 2.08) | 0.16 | 1.37 (0.90; 2.10) | 0.15 |
| CERAD score | 0.65 (0.42; 1.01) | 0.06 | 0.59 (0.36; 0.97) | 0.04 | 0.58 (0.35; 0.96) | 0.03 |

*African ancestry was considered present if equal to or greater than 2% of African ancestry.

OR: odds ratio

BB: Braak & Braak; CERAD: Consortium to Establish a Registry for Alzheimer's disease.

Model 1: Ordinal logistic regression model adjusted for age, sex, and education.

Model 2: Ordinal logistic regression model adjusted for age, sex, education, and *APOE4* status.

**Supplementary Table 4. Association of Informant Questionnaire on Cognitive Decline in the Elderly (IQCODE) with quantitative proportions of African ancestry and AD neuropathological burden (n=400)**

|  | Model 1 |  | Model 2 |  | Model 3 |  |
| --- | --- | --- | --- | --- | --- | --- |
|  | **β (95% CI)** | **p** | **β (95% CI)** | **p** | **β (95% CI)** | **p** |
| AFR | 0.01 (-0.00; 0.03) | 0.17 | 0.01 (-0.00; 0.03) | 0.19 | 0.01 (-0.01; 0.03) | 0.25 |
| BB stage |  |  |  |  |  |  |
| *III-IV* | 0.07 (-0.04; 0.18) | 0.23 | 0.04 (-0.07; 0.17) | 0.46 | 0.04 (-0.08; 0.16) | 0.48 |
| *V-VI* | 0.77 (0.56; 0.97) | **<0.001** | 0.75 (0.54; 0.95) | **<0.001** | 0.75 (0.54; 0.96) | **<0.001** |
| CERAD score | |  |  |  |  |  |
| *Moderate* | 0.24 (0.10; 0.38) | **0.001** | 0.21 (0.07; 0.36) | **0.004** | 0.20 (0.06; 0.35) | **0.007** |
| *Frequent* | 0.54 (0.36; 0.72) | **<0.001** | 0.50 (032; 0.69) | **<0.001** | 0.48 (0.29; 0.67) | **<0.001** |

AFR: global African ancestry (continuous 10% increments); BB: Braak & Braak; CERAD: Consortium to Establish a Registry for Alzheimer's disease

Model 1: Linear regression model including African Ancestry, Braak & Braak stage, and CERAD score

Model 2: Linear regression model including African Ancestry, Braak & Braak stage, and CERAD score adjusted for age, sex, and education

Model 3: Linear regression model including African Ancestry, Braak & Braak stage, and CERAD score adjusted for age, sex, education, and APOE4

**Supplementary Table 5. Association of Clinical Dementia Rating sum of boxes (CDR-SOB) with African ancestry (AFR) and Alzheimer's disease (AD) pathology considering an interaction between AFR and AD**

|  | β | 95% CI | P |
| --- | --- | --- | --- |
| Interaction between AFR and Braak stage | | | |
| AFR | -0.001 | -0.020; 0.018 | 0.91 |
| Braak stage |  |  |  |
| *III-IV* | 1.139 | -0.128; 2.405 | 0.08 |
| *V-VI* | 8.139 | 6.393; 9.885 | <0.001 |
| AFR*Braak stage^†^ |  |  |  |
| *AFR*III-IV* | 0.008 | -0.028; 0.044 | 0.668 |
| *AFR*V-VI* | 0.969 | 0.043; 0.151 | <0.001 |
| Interaction between AFR and CERAD score | | | |
| AFR | 0.0001 | -0.018; 0.019 | 0.99 |
| CERAD score |  |  |  |
| *Moderate* | 2.183 | 0.554; 3.812 | 0.009 |
| *Frequent* | 6.865 | 5.201; 8.530 | <0.001 |
| AFR*CERAD score^┤^ |  |  |  |
| *AFR*Moderate* | 0.016 | -0.033; 0.064 | 0.53 |
| *AFR*Frequent* | 0.077 | 0.029; 0.126 | 0.002 |

^†^p for trend for the interaction between AFR and Braak stage=0.002

**^┤^** p for trend for the interaction between AFR and CERAD score=0.007

**Supplementary Table 6**. **Association between AD neuropathological burden and functional cognitive scores with one or two non-European *APOE* alleles (local APOE ancestry) in *APOE4-* individuals (n=235, see Figure 1 for grouping criteria)**

|  |  | Model 1 (without AFR adjustment) | | Model 2 (with AFR adjustment) | |
| --- | --- | --- | --- | --- | --- |
| Outcomes | **Pathology adjustments** | **β (95% CI)** | **p** | **β (95% CI)** | **p** |
| BB | NA | -0.12 (-0.46; 0.22) | 0.493 | -0.23 (-0.63; 0.17) | 0.263 |
| CERAD | NA | -0.27 (-0.48; -0.05) | **0.015** | -0.28 (-0.53; -0.02) | **0.032** |
| IQCODE | NA | 0.03 (-0.08; 0.14) | 0.60 | 0.04 (-0.10; 0.18) | 0.55 |
|  | BB+CERAD | 0.09 (-0.01; 0.19) | 0.08 | 0.11 (-0.01; 0.23) | 0.07 |
|  | BB | 0.04 (-0.06; 0.15) | 0.41 | 0.06 (-0.06; 0.18) | 0.33 |
|  | CERAD | 0.10 (-0.00; 0.20) | 0.06 | 0.12 (-0.00; 0.24) | 0.05 |

Reference: Individuals EUR APOE4- alleles (genotypes 22, 23 or 33) (n=138)

AFR: global African ancestry (continuous 10% increments); BB: Braak & Braak; CERAD: Consortium to Establish a Registry for Alzheimer's disease; IQCODE: Clinical Dementia Rating sum of boxes, NA: not applicable

Model 1: Linear regression model adjusted for age, sex, education, and AD neuropathology when indicated.

Model 2: Linear regression model adjusted for age, sex, education, AD neuropathology when indicated, and AFR.

**Supplementary Table 7. Association of AD neuropathological burden and functional cognitive scores with local European or non-European *APOE*4+ ancestries using corresponding ancestries of *APOE*4- individuals as references**

| (A) EUR *APOE*4+ local ancestry (n=31)* | | Model 1 (no AFR adjustment) | | Model 2 (with AFR adjustment) | |
| --- | --- | --- | --- | --- | --- |
| Outcomes | **Pathology adjustments** | **β (95% CI)** | **p** | **β (95% CI)** | **p** |
| BB | NA | 0.52 (0.01; 1.03) | **0.048** | 0.51 (-0.002; 1.03) | 0.051 |
| CERAD | NA | 0.61 (0.28; 0.95) | **<0.001** | 0.61 (0.27; 0.95) | **<0.001** |
| CDR-SOB | NA | 0.15 (-0.04; 0.34) | 0.11 | 0.16 (-0.03; 0.35) | 0.10 |
|  | BB+CERAD | 0.07 (-0.10; 0.25) | 0.43 | 0.07 (-0.10; 0.25) | 0.40 |
|  | BB | 0.12 (-0.06; 0.29) | 0.19 | 0.12 (-0.05; 0.30) | 0.17 |
|  | CERAD | 0.04 (-0.14; 0.22) | 0.65 | 0.05 (-0.13; 0.22) | 0.59 |
| (B) Non-EUR *APOE*4+ local ancestry (n=17)** | | **Model 1 (no AFR adjustment)** | | **Model 2 (with AFR adjustment)** | |
| BB | NA | -0.21 (-1.08; 0.65) | 0.62 | -0.21 (-1.10; 0.67) | 0.62 |
| CERAD | NA | 0.01 (-0.52; 0.70) | 0.76 | 0.09 (-0.51; 0.69) | 0.76 |
| CDR-SOB | NA | 0.05 (-0.25; 0.35) | 0.74 | 0.05 (-0.25; 0.35) | 0.74 |
|  | BB+CERAD | 0.15 (-0.14; 0.45) | 0.30 | 0.15 (-0.15; 0.46) | 0.31 |
|  | BB | 0.15 (-0.13; 0.42) | 0.29 | 0.14 (-0.13; 0.42) | 0.30 |
|  | CERAD | 0.09 (-0.19; 0.38) | 0.52 | 0.09 (-0.20; 0.38) | 0.54 |

*Reference for (A): Individuals *APOE4-* (genotypes 22, 23, or 33) with local European ancestries (n=138)

** Reference for (B): Individuals APOE4- (genotypes 22, 23 or 33) with local non-European ancestries (n=24)

AFR: African ancestry (continuous 10% increments); BB: Braak & Braak; CERAD: Consortium to Establish a Registry for Alzheimer's disease; NA: not applicable

Model 1: Linear regression model adjusted for age, sex, education, and AD neuropathology when indicated.

Model 2: Linear regression model adjusted for age, sex, education, AD neuropathology when indicated, and AFR.

OBS. Only individuals homozygous for *APOE* local ancestry were included
